# Two ethical concerns about the use of persuasive technology for vulnerable people

**DOI:** 10.1111/bioe.12683

**Published:** 2019-10-15

**Authors:** Naomi Jacobs

**Affiliations:** ^1^ Technische Universiteit Eindhoven The Netherlands

**Keywords:** ethics, health and wellbeing, health‐related behaviour change, persuasive ethics, persuasive technology, vulnerable people

## Abstract

Persuasive technologies for health‐related behaviour change give rise to ethical concerns. As of yet, no study has explicitly attended to ethical concerns arising with the design and use of these technologies for *vulnerable* people. This is striking because these technologies are designed to help people change their attitudes or behaviours, which is particularly valuable for vulnerable people. Vulnerability is a complex concept that is both an ontological condition of our humanity and highly context‐specific. Using the Mackenzie, Rogers and Dodds’ taxonomy of vulnerability, this paper identifies (a) the wrongs or harms to which a person is vulnerable, (b) the source of this vulnerability, and (c) the safeguards needed in response. Two ethical concerns with the design of persuasive technology for vulnerable people are discussed: the concerns of taking into account users' interests and their autonomy.

## 1 INTRODUCTION

Behaviours that pose health risks, such as smoking, overeating and physical inactivity, contribute significantly to the development of various serious chronic diseases,1WHO. (2015). *World Health Organization fact sheet no. 310: The top 10 causes of death*. Retrieved from http://www.who.int/mediacentre/factsheets/fs310/en/ [Accessed Jun 26, 2019]. affecting personal wellbeing as well as indirectly adding to high healthcare costs. Persuasive technologies for health‐related behaviour change have been proposed as a way to help people reduce or eliminate these behaviours.

Persuasive technology (hereafter PT) is a class of technologies that are designed to change a person's attitude, behaviour, or both.2IJsselsteijn, W., de Kort, Y., Midden, C., Eggen, B., & van den Hoven, E. (2006). Persuasive technology for human well‐being: Setting the scene. In W. A. IJsselsteijn, Y. A. W. de Kort, C. Midden, B. Eggen, & E. van den Hoven (Eds.), *Persuasive Technology. PERSUASIVE 2006. Lecture Notes in Computer Science* (Vol. 3962, pp. 1–5). Heidelberg, Berlin, Germany: Springer. Fogg, B. J. (2003). *Persuasive technology: Using computers to change what we think and do*. San Francisco, CA: Morgan Kaufmann. What is important is that the standard definition of PT by Fogg prescribes that PTs always bring about a *voluntary* change of behaviour or attitude.3Fogg, op. cit. Although force and misleading information or dishonest communication may persuade as well, the definition of PT by Fogg that is endorsed in this article explicitly excludes instances of influence by coercion or manipulation from the realm of PT. The exact difference between persuasion, manipulation and coercion is discussed in Section 2, as is how to ensure that persuasion by PT excludes any instances of coercion or manipulation.

Furthermore, this article focuses on cases in which PT is used to persuade persons to act in their best interests and in alignment with their personal goals (e.g. to eat more healthily). Cases in which persons are persuaded by PT to act in a manner not in line with their own interest (e.g. to spend money on products that they do not need) are explicitly excluded from the scope of this paper. The paper focuses on instances of persuasion by PT in which a user would reasonably consent both to the *ends* of the persuasion (i.e. the target behaviour of the PT) and to the *means* of the persuasion (i.e. the persuasive tools used by the PT to persuade the user to perform the target behaviour). This is discussed in more detail in Sections 2 and 4.4.

PTs for health and wellbeing are aimed at health promotion or disease management.4Orji, R., & Moffatt, K. (2016). Persuasive technology for health and wellness: State‐of‐the‐art and emerging trends. *Health Informatics Journal*, *24*(1), 66–91. Examples include HAPIfork, a smart fork that monitors and tracks the user’s eating habits and aims to persuade users to eat more slowly; MyFitnessPal, a calorie‐counter and diet‐tracker app, which persuades users to eat more healthily and to exercise more; Sobriety Counter, an app that persuades users to stop drinking alcohol via motivational features such as visualizing the money a user saves by not drinking or providing scientific health statistics about the user's body and how it improves without alcohol; the GlowCap, a persuasive medication‐adherence technology that consists of a smart pill bottle and cap that flashes an orange light when a user should take her medication; and MySugr, a diabetes‐management app that tracks users' blood sugar and provides them with personal diabetes coaching.

The use of such PTs can give rise to multiple ethical concerns. As of yet, however, no study in the field of persuasive ethics has explicitly focused on the ethical concerns that arise with the design and use of PTs for *vulnerable* people.5Berdichevsky, D., & Neuenschwander, E. (1999). Towards an ethics of persuasive technology. *Communications of the ACM, 42*(5), 51–58; Davis, J. (2009). Design methods for ethical persuasive computing, In *Proceedings of the 4th International Conference on Persuasive Technology*, ACM, Claremont, California; Yetim, F. (2011). A set of critical heuristics for value sensitive designers and users of persuasive systems. In *ECIS 2011 Proceedings, Helsinki*; Spahn, A. (2011). And lead us (not) into persuasion...? Persuasive technology and the ethics of communication. *Science and Engineering Ethics, 18*(4), 633–650; Smids, J. (2012). The voluntariness of persuasive technology. In M. Bang & E. L. Ragnemalm (Eds.). *Persuasive Technology. Design for Health and Safety. PERSUASIVE 2012. Lecture Notes in Computer Science* (vol. 7284, pp. 123–132). Heidelberg, Berlin, Germany: Springer; Burri Gram‐Hansen, S. (2009). Towards an approach to ethics and HCI development based on Løgstrup’s ideas. *INTERACT '09 Proceedings of the 12th IFIP TC 13 International Conference on Human‐Computer Interaction: Part I* (pp. 200–203). Uppsala, Sweden. This is striking because PTs are designed to help people change their attitudes or behaviours—something that is often particularly valuable for vulnerable people in order to help them cope better with their vulnerabilities. If people cope with their vulnerabilities, this could mean that they avoid the consequent harm that is likely because of their vulnerability, or that the degree of their vulnerability is itself diminished.6I want to thank an anonymous reviewer for pointing out this distinction. With regard to PT, an example of the first instance is the GlowCap—a PT that helps people who are vulnerable to forgetfulness to adhere to their medication regimen. An example of the second instance is an app that aims to reduce people's vulnerability to sexual assault by providing them with exercises to become better at self‐defence. Ideally, the degree of the vulnerability to sexual assault itself will be lessened over time with the help of the PT.

In this article, I will argue for the need to address the ethical concerns that arise from the design and implementation of PTs for vulnerable people, highlighting specific ethical concerns and developing suggestions for ways to deal with them. The article proceeds as follows. First, the notion of PT is delineated. Second, the concept of vulnerability is clarified, and I discuss its ethical significance in relation to PT for health and wellbeing. Lastly, I discuss two ethical concerns that arise from the design and use of PTs from a vulnerability perspective.

## 2 PERSUASIVE TECHNOLOGY

PTs make use of persuasion, but what exactly is *persuasion*? In the predominant typology of influence used in the bioethics literature, persuasion is often understood as rational persuasion, meaning influence by reason and argument.7Blumenthal‐Barby, J. S. (2012). Between reason and coercion: Ethically permissible influence in health care and health policy contexts. *Kennedy Institute of Ethics Journal, 22*(4), 345–366. Beauchamp and Childress, for instance, state that in persuasion ‘a person must come to believe in something through the merit of reasons another person advances’.8Beauchamp, T. L., & Childress, J. F. (2013). *Principles of biomedical ethics*. Oxford, U.K.: Oxford University Press, p. 139. Rational persuasion is distinguished in this dominant typology from coercion (influence by force, depriving the coerced person of choice entirely) and from manipulation (meaning everything between rational persuasion and coercion).9Blumenthal‐Barby, op. cit.


'Manipulation' in particular is a broad and underdeveloped category in this typology, and Blumenthal‐Barby has rightly pointed out that this traditional tripartite categorization of influence used in bioethics literature is ‘in desperate need of conceptual refining and ethical analysis’.10Blumenthal‐Barby, op. cit., p. 345


In order to conceptually refine the categories of persuasion, coercion and manipulation, I propose the following definitions of each category. I suggest letting go of the narrow understanding of persuasion as being solely rational, and instead understand persuasion in a much broader sense that includes both rational and non‐rational means of influence. Non‐rational means of influence include, for example, framing, setting up defaults, changing choice architecture, playing on emotions, appealing to authority, and peer pressure.11Petty, R. E., & Cacioppo, J. T. (1986). The elaboration likelihood model of persuasion. In *Communication and persuasion* (pp. 1–24). New York, NY: Springer; Thaler, R. H., & Sunstein, C. R. (2008) *Nudge*. New Haven, CT: Yale University Press; Blumenthal‐Barby, op. cit. Of crucial importance is that persuasion, both by rational and by non‐rational means, never significantly blocks or burdens options; furthermore, a person is aware of the fact that she is being intentionally influenced and is aware of the mechanisms of that influence, and the influence is in the best interests and is in alignment with the personal goals of the person being influenced.12These three criteria are based upon criteria identified by Thaler, R. H. (2015). The power of nudges, for good and bad. *New York Times*. Retrieved from https://www.nytimes.com/2015/11/01/upshot/the-power-of-nudges-for-good-and-bad.html [Accessed Jun 8, 2019] on what constitutes a morally permissible nudge. And upon the criteria identified by Blumenthal‐Barby, op.cit. when autonomy and nonargumentative influence are compatible.


Manipulation is best understood as defined by Susser et al., namely as ‘imposing a hidden or covert influence on another person's decision‐making’.13Susser, D., Roessler, B., & Nissenbaum, H. (2019). Technology, autonomy, and manipulation. *Internet Policy Review*, 8(2). The most important distinction between persuasion and manipulation is that the latter ‘disrupts the target's capacity for self‐authorship’;14Ibid. that is, someone is being influenced in a way that thwarts their capacity to become aware of this influence. Coercion, lastly, is to be understood as influencing someone by irresistible treats, depriving someone of choice entirely.15Blumenthal‐Barby, op. cit.; Susser et al., op. cit.


In standard ethical analysis, coercion is almost always impermissible, except when someone forms a threat to self or others.16Blumenthal‐Barby, op. cit. Manipulation, in which a person's decision‐making power is covertly subverted and their autonomy is undermined, is always ethically impermissible. Whether persuasion is ethically permissible or not depends on the following two issues: (a) whether the persuasion happens in alignment with a person's own goals and whether the PT sufficiently takes into account a person's interests and needs (this issue is discussed further in Section 4.3); and (b) whether the instance of persuasion thwarts a person's autonomy (this issue is discussed in detail in Section 4.4).

Lastly, one must note that persuasion by technology is different from persuasion by humans in several important ways. Firstly, technology does not inherently possess the ability to respond to a human being in the same way that another human being would. This necessitates an explicit consideration of what kinds of responses are desirable. In addition, technology is inherently persistent: a computer does not get tired, discouraged or frustrated like humans do. Furthermore, technology is ubiquitous and may have access to people's most private locations, like the bedroom or bathroom, that a human persuader would not be allowed to enter.17Fogg, op. cit. Technology thus changes the game of persuasion, giving rise to new ethical challenges.

## 3 DIMENSIONS OF VULNERABILITY

In the bioethics literature, vulnerability has often been conceptualized in two distinct ways: either (a) as an ontological condition of humanity, or (b) as a marker for context‐specific needs.

The first view, that vulnerability is an ontological condition of humanity, entails that vulnerability is a universal, inevitable and enduring aspect of the human condition, namely to be fragile and susceptible to wounding and suffering.18Fineman, M. A. (2008). The vulnerable subject: Anchoring equality in the human condition. *Yale Journal of Law and Feminism, 20*(1), 1–23. However, the problem with such an ontological conception of vulnerability is that it obscures rather than enables the identification of the context‐specific needs of particular groups or individuals within populations at risk. Such a conceptualization of vulnerability is not useful when we aim to identify the specific ethical concerns that may arise with PT for particular groups or individuals.

In response to this ontological view of vulnerability, others have in contrast conceptualized vulnerability as a marker to identify specific persons or groups that require extra attention and care.19Levine, C., Faden, R., Grady,C., Hammerscmidt,D., Eckenwiler, L., & Sugarman, J. (2004). The limitations of 'vulnerability' as a protection for human research participants. *American Journal of Bioethics, 4*(3), 44–49. Luna, F. (2009). Elucidating the concept of vulnerability: Layers not labels. *International Journal of Feminist Approaches to Bioethics, 2*(1), 121–139. This approach to vulnerability is more useful when we aim to identify the special needs of vulnerable people with regard to the design and use of PT. However, the labelling of specific people or groups as vulnerable can create another problem, because it might lead to unwarranted and unjust paternalistic responses, stereotyping, disqualification, or discrimination.20Mackenzie, C., Rogers, W., & Dodds, S. (Eds.). (2013). *Vulnerability: New essays in ethics and feminist philosophy*. Oxford, U.K.: Oxford University Press. Meek Lange, M., Rogers, W., & Dodds, S. (2013). Vulnerability in research ethics: A way forward'. *Bioethics, 27*(6), 333–340.


Instead of endorsing one of the two abovementioned conceptualizations of vulnerability, this article endorses a third view that reconciles the two previous views on vulnerability in a new, overarching conceptualization. This third view on vulnerability is that of Mackenzie, Rogers and Dodds,21Mackenzie et al., op. cit. who have developed a taxonomy of vulnerability.

Mackenzie, Rogers and Dodds22Ibid. have reconciled the ontological conception of vulnerability with the conception of vulnerability as a marker to identify specific persons and groups that require extra attention and care in a taxonomy of vulnerability. This taxonomy distinguishes different sources and states of vulnerability and enables a fine‐grained analysis of the sense in which vulnerability is both an ontological condition of our humanity and a context‐specific phenomenon.23Ibid. Furthermore, the taxonomy clarifies how vulnerability affects a wide range of people in one way or another, and identifies which of them have specific needs. The taxonomy is powerful because it helps to identify clearly (a) the wrong or harm a person is vulnerable to, (b) the source of this vulnerability, and (c) the safeguards that are needed in response.

The taxonomy distinguishes two *states* of vulnerability: vulnerability can be *dispositional* or *occurent*. In addition, there are different *sources* of vulnerability: *inherent* and *situational*. Inherent sources of vulnerability are intrinsic to the human condition; they arise from our corporeality and our affective and social natures.24Mackenzie, C., Rogers, W., & Dodds, S. (2013). Introduction: What is vulnerability, and why does it matter for moral theory? In C. Mackenzie, W. Rogers & S. Dodds (Eds.) *Vulnerability: New essays in ethics and feminist philosophy* (pp. 1–29). Oxford, U.K.: Oxford University Press. Situational sources of vulnerability, on the other hand, are context‐specific. These sources are caused, or exacerbated, by the social, political, economic, or environmental context that a person or social group is in.25Ibid. p. 9. There is also a particular subset of situational sources, namely *pathogenic* vulnerabilities. Pathogenic vulnerabilities can arise when a response intended to ameliorate vulnerability has the paradoxical effect of exacerbating existing vulnerabilities or creating new ones.26Ibid. For example, people with cognitive disabilities who are vulnerable because of their care needs are susceptible to pathogenic forms of vulnerability such as emotional or physical abuse by their caregivers.

Sources of vulnerability are problematic when they cause a person or a group of persons to experience a diminished capacity to meet or protect their needs or safeguard their interests, putting them at increased risk of suffering harm or wrong.

## 4 PERSUASIVE ETHICS FROM A VULNERABILITY PERSPECTIVE

To indicate the various ethical concerns that could arise for a vulnerable person from the use of PT for health‐related behaviour change, I discuss two examples of such technologies used by persons who are vulnerable in distinct ways. The first example concerns a persuasive medication‐adherence technology that is used by an older woman. The second example concerns a calorie‐counter app that aims to persuade users to lose weight and is used by a young woman with an eating disorder.

### 4.1 Miriam and the GlowCap

The Vitality GlowCap is a persuasive ‘medication adherence system comprised of a smart cap and bottle’.27Retrieved from https://nanthealth.com/vitality/ [Accessed Jan 20, 2019]. It is designed such that ‘automated visual and audible alerts during scheduled dosage windows signal that it is time for the user to take his or her medications’;28Ibid. it thus intervenes at the right time, and makes use of the persuasive tool of suggestion. Furthermore, the GlowCap makes use of the persuasive tools of self‐tracking and surveillance: the user has access to an online portal where she can view her own progress report on how well she adhered to her medication regimen. The company behind the PT can also send this online progress report to clinicians, care‐managers, and family or friends at the primary user's behest, who can subsequently act on the information provided by the report.

Imagine Miriam, an 88‐year‐old woman who lives independently at her home. Miriam needs to take various medicines each day, including ones to lower her bad cholesterol, manage her high blood pressure, and deal with osteoporosis. However, Miriam has trouble remembering when she needs to take her medication. The GlowCap can assist Miriam to adhere to her medication schedule, which ultimately helps Miriam to live independently at home. However, alongside this advantage there are also serious concerns attached to the use of the GlowCap for a person such as Miriam that might cause her to become more vulnerable than she was initially. As mentioned, the GlowCap makes use of the persuasive tool of surveillance by creating an online progress report on how well Miriam adheres to her medication schedule, and the company behind the GlowCap can share this online report with clinicians, care‐managers, and family or friends at the primary user's behest, who can subsequently act on the information provided by the report. Owing to her age, Miriam relies on the care and support of family members and caregivers, and this reliance could make Miriam reluctant to refuse granting those caregivers and family members access to her online data that is gathered by the GlowCap. But being surveilled by family and caregivers diminishes Miriam’s privacy, and the realization that her family and caregivers might act upon the information that is gathered in her online progress report (e.g. to decide that Miriam is no longer able to live independently at home but needs to go to a nursing home, even if this is against Miriam’s own wishes) could make Miriam feel more powerless than she felt before. Thus, although the GlowCap aims to ameliorate Miriam's medication adherence, it might have the negative effect of making her feel more powerless than she felt before. Without proper attention to the complexities of vulnerability, a technology designed with the intent to ameliorate certain vulnerabilities may instead become an additional pathogenic source of vulnerability.

### 4.2 Elisa and MyFitnessPal

Imagine Elisa, a woman in her early twenties who struggles with exercise bulimia.29Exercise bulimia is a subset of the eating‐disorder bulimia, in which a person is compelled to exercise in an attempt to burn calories to an excessive level that negatively affects their health. Retrieved from https://en.wikipedia.org/wiki/Exercise_bulimia [Accessed Jun 26, 2019]. Elisa uses MyFitnessPal, a calorie‐counting app that allows users to track and input their daily food intake, provides feedback on the number of calories and nutrients needed per day, allows users to set weight and nutrient goals, and provides advice on how to reach these goals.30Levinson, C. A., Fewell, L., & Brosof, L. C. (2017). My Fitness Pal calorie tracker usage in the eating disorders. *Eating Behaviors, 27*, 14–16. Elisa derives her self‐worth to a great extent from what others think of her and believes that she has to meet certain beauty ideals to be accepted by others. Thus Elisa suffers from a situational source of vulnerability, which makes her susceptible to exercise bulimia. This susceptibility to exercise bulimia is an occurent and inherent vulnerability for Elisa.

The app MyFitnessPal is designed to help users obtain and maintain healthy weight goals; however, research has shown that it ‘is widely used in an eating disorder population and is perceived as contributing to eating disorder symptoms’.31Ibid.: 14. MyFitnessPal makes use of the persuasive tool of conditioning: it rewards users with trophies or badges for receiving certain health‐related goals, and enables competition with other users by comparison with the achievements of others using the app. This community component can be especially triggering for people suffering from an eating disorder (ED), as these are competitive illnesses.32Sharkey, L. (2018). 'A twisted comparison game': How fitness apps exacerbate eating disorders. Retrieved from https://broadly.vice.com/en_us/article/pammjn/a-twisted-comparison-game-how-fitness-apps-exacerbate-eating-disorders [Accessed Jun 26, 2019]. The persuasive tool of conditioning and its competition component could push driven eating‐disorder sufferers to under‐eat or fast for longer and more often.33Ibid. A real‐life ED sufferer explained in an interview that ‘having an app telling me how far I’ve gone just spurs me on to want to fast more’.34Ibid. Another real‐life ED sufferer testified in the same interview that it could become a compulsion to keep track of their calorie intake and to log in multiple times a day to add every detail of their diet to the app, such that it changed ‘what I would eat and plan to eat so that it would always be within my “acceptable” calorie range’.35Ibid. For individuals who do not have a dysfunctional relationship with food, an app like MyFitnessPal can provide the data and structure that is needed to meet health‐related target behaviours. For others, like Elisa, it can exacerbate situational and inherent sources of vulnerability and support, or even encourage, unhealthy and harmful behaviours, with potentially long‐lasting physical and mental consequences.

The examples of Miriam and Elisa highlight various ethical concerns that might arise with the use of PT by people who are vulnerable. There are two concerns in particular that need close examination, and I discuss them below.

### 4.3 The concern of taking into account users' interests

A concern that clearly comes to the fore in the examples of Miriam's use of the GlowCap and Elisa's use of MyFitnessPal is that the design of these PTs is not adequately informed by the experiences, interests and needs of its users. In the case of Miriam, the data‐sharing function of the GlowCap did not adequately take her autonomy into account and made her feel powerless. In the example of Elisa, it became clear that the design of a diet app like MyFitnessPal is not informed by the experiences of users who struggle with body image problems and eating disorders.

In order to avoid the situation in which a technology takes the needs and interests of users insufficiently or inadequately into account, technology design should be informed by the experiences, interests and needs of prospective users. The aim is to ensure that technology design is inclusive and to avoid mistaken assumptions about how the technology will be used arising from not taking into account the likely diversity of users. To ensure that the interests and needs of (vulnerable) users are taken into account, designers have to elicit the needs of their prospective users during the design process while keeping two crucial aspects in mind, as put forward by Pommeranz et al.: ‘(1) taking real life contexts into account and (2) supporting communication between stakeholders and designers’.36Pommeranz, A., Detweiler, C., Wiggers, P., & Jonkers, C. (2012). Elicitation of situated values: Need for tools to help stakeholders and designers to reflect and communicate. *Journal of Ethics and Information Technology*, *14*(4), 285–303.


What goes wrong when designers fail to take into account these two crucial aspects becomes clear when we take a critical look at the 'golden rule' for PT design that was developed by Berdichevsky and Neuenschwander in their influential article on the ethics of PT.37Berdichevsky, D., & Neuenschwander, E. (1999). Towards and ethics of persuasive technology. *Communications of the ACM, 42*(5), 51–58. Their golden rule prescribes that, as a designer, you should never do to others what you don't want to be done to you. For this golden rule to be meaningful, however, a designer needs to assume that everyone is more or less like her, while in reality they are not. When you design for people who, as a result of their inherent or situational vulnerabilities, do not have the same needs or interests as you do, how does the golden rule guarantee that you as a designer create what is best not only for yourself but also for *others*?

The pitfall is that designers compile an idealized person that is based on their own experiences, needs and preferences and take that as their vantage point. Thus they do not take real life contexts adequately into account, and vulnerable users who differ from this idealized vantage point do not receive sufficient attention and provision in the technology design. This pitfall clearly occurs with the design of MyFitnessPal, which is aimed at 'ideal' individuals who have a functional relationship with food and which fails to provide effective attention and provision to vulnerable users who have a dysfunctional relationship with food.

Another related pitfall is that designers create interventions aimed at ameliorating the inherent or situational vulnerabilities of their intended prospective users, but that these interventions have instead the opposite effect of creating or exacerbating vulnerability, for instance by (unintentionally) undermining the autonomy of the user or by increasing their sense of powerlessness, as we saw with Miriam and the data‐sharing function of the GlowCap. In order to avoid this pitfall, it is crucial that there are adequate tools that support communication between designers and stakeholders on the values, needs and interests important to the users.

Taking real life contexts into account and supporting communication between designers and users can, to a large extent, avoid these pitfalls. When the design of PT is informed by the experiences, needs and interests of the (vulnerable) people the technology is designed for, the pitfalls of unintentionally creating or exacerbating vulnerability by design, or the risk of providing vulnerable users with ineffective attention and provision in the design are minimized. To make sure that a design is indeed informed by the experiences, needs and interests of the (vulnerable) people the technology is designed for, we need adequate elicitation tools. The problem is, however, that there is a lack of elicitation tools that support a shared understanding of interests between stakeholders and designers, and that support self‐reflection by stakeholders.38Pommeranz, A., Detweiler, C., Wiggers, P., & Jonkers, C. (2012). Elicitation of situated values: Need for tools to help stakeholders and designers to reflect and communicate. *Journal of Ethics and Information Technology*, *14*(4), 285–303. Furthermore, there exist great personal differences regarding which method for expressing needs and interests works best.39Ibid. This is a problem in general, but it is an even bigger problem for vulnerable people, who are often already less able to indicate their needs or protect their interests. Thus, what we are in urgent need of is further research on elicitation tools that work well for vulnerable people.40There are promising approaches to elicitation tools, for example the iRequire Approach, a tool‐supported approach for end‐user‐led requirement elicitation based on reported needs, developed by Norbert Seyff, Florian Graf and Neil Maiden (2010). However, such an approach does not pay specific attention to the needs of *vulnerable* people.


### 4.4 The concern of manipulation and coercion

To respect a person's autonomy is to acknowledge a person's right to hold views, make choices and take actions that are based on their own values and beliefs.41Beauchamp, T. L., & Childress, J. F. (2013). *Principles of biomedical ethics*. Oxford, U.K.: Oxford University Press. p. 106. Respect for personal autonomy is an important value, especially in responses to vulnerability. This is, as Mackenzie points out, because respect for autonomy can ‘counter the sense of powerlessness and loss of agency that is often associated with vulnerability’42Mackenzie, C. (2013). The importance of relational autonomy and capabilities for an ethics of vulnerability. In C. Mackenzie, W. Rogers & S. Dodds (Eds.) *Vulnerability: New essays in ethics and feminist philosophy* (pp. 33–59). Oxford, U.K.: Oxford University Press. and because respect for autonomy can counter the risks of coercion and manipulation.

The problem with manipulation (i.e. imposing a hidden influence on another person's decision‐making and thereby infringing upon their autonomy) and coercion (i.e. influencing someone by irresistible treats and depriving someone of choice entirely) is that it has corrosive effects on a person's autonomy. In order to protect people's autonomy, instances of manipulation and coercion should be explicitly excluded from the realm of PT.

However, the boundary lines between coercion, manipulation and persuasion are not always clear‐cut. As already discussed in Section 2, for PT to exclude instances of manipulation or coercion and to respect a person's autonomy, it must meet the following three criteria: (a) a PT may never significantly block or burden a user's options, (b) a PT must make its user aware of the fact that she is being intentionally persuaded and make her aware of the tools of persuasion, and (c) a PT must persuade a person in alignment with her own personal goals, i.e. a person must share the targeted behavioural outcome of the PT.43These three criteria are based upon criteria identified by Thaler, op. cit. on what constitutes a morally permissible nudge, and on the criteria identified by Blumenthal‐Barby, op. cit. when autonomy and nonargumentative influence are compatible. Thus, for example, with a PT that aims to persuade users to exercise more, a user must share that target behaviour and should be aware that the PT will make use of persuasive tools such as sending encouraging messages at the right time and giving rewards for achievements in order to persuade her to exercise more. Also, she should have options to choose other than the PT suggests or to stop using the PT altogether.

For vulnerable people who may, owing to various inherent, situational or pathogenic sources of vulnerability at play, have a diminished capacity to protect their needs and safeguard their interests, it is especially important that a PT meets these three criteria and that the technology does not thwart their autonomy. But how can we ensure that a PT meets these three criteria, that users' autonomy is respected, and that instances of manipulation and coercion are excluded? This can be ensured by a valid consent procedure for PT.

What exactly constitutes a valid consent procedure for PT is a question that requires an extensive analysis and that exceeds the scope of this article. What I provide here is an outline of what a consent procedure for PT should look like.

To start with, it must be clear exactly *to what* users of a PT are consenting. I distinguish four aspects of PTs to which users can consent. First, there are the goals and intended behavioural outcomes of a PT, for example better medication adherence, losing weight, exercising more or drinking less alcohol. Secondly, there are the persuasive tools that a PT utilizes. Persuasive tools are the strategies that a PT applies to change a user's attitude or behaviour. Examples are the persuasive tool of reduction (reducing complex behaviour to simple tasks can influence users to perform the desired behaviour), or the persuasive tool of self‐monitoring (by letting users track their own performance they know how well they are performing the target behaviour, which increases the likelihood that they will continue).44Fogg op. cit. Thirdly, there are the types of individual interactions of the PT with the user through the use of the technology. This can, for example, be a tailored text message or a blinking light that indicates that this is the opportune moment to perform the target behaviour. The fourth aspect that users need to consent to is the use and storage of their data by the company behind the PT.45I want to thank an anonymous reviewer for pointing out this required fourth aspect. In order to ensure valid consent, a user should give consent to *all four* aspects of a PT.46This is similar to what is sometimes referred to as 'separate granular consent options' in relation to the conditions for consent in the European General Data Protection Regulation (GDPR).


We can account for valid consent via design requirements that make it easy for the user to give consent on all four aspects. What I propose is that design requirements for PTs are informed by Onora O'Neill's conception of consent, because O'Neill has come up with a relatively simple conception of consent that appears to be well suited to the context of PT.47O'Neill, O. (2003). Some limits of informed consent. *Journal of Medical Ethics, 29*, 4–7. O'Neill has argued that the practice of consent should be understood as a way to prevent someone from being manipulated or coerced. This is ensured when someone is given a limited amount of accurate and relevant information (thereby ensuring that they are not overwhelmed by information) and is provided with user‐friendly ways to extend this amount (thereby ensuring that they are not manipulated). There should also be easy ways of rescinding consent once given, thereby ensuring that the person is not coerced.

O'Neill's conception of consent can be translated into design requirements for PT. Consider the following, somewhat simplified, example of an app that aims to persuade users to drink less alcohol. For such a persuasive app, in order to ensure valid consent from its users, the design requirements for consent should look something like this: a simple user interface where the four aspects of the PT are clearly distinguished. When the user clicks on 'intended behavioural outcome', she sees the target behaviour clearly explained, in this case alcohol‐intake reduction. The user than sets a specific goal, for example a maximum of 2 units of alcohol per week. Subsequently, the user needs to answer 'Do you consent to this intended outcome?', with the options 'yes/no/more info'. After ticking on the option 'more info', the user is provided with more detailed information on the intended behavioural outcome and is again presented with the options yes/no. The same mechanism holds for aspect two, where the user is asked, for example, 'Do you consent to the use of the persuasive tool of self‐monitoring, which consist of self‐tracking the amount of alcohol you take in per day?', again with the options 'yes/no/more info'. For the third aspect, namely the sort of individual interactions, the user has to answer the question 'Do you consent to the app sending you a maximum of five text messages throughout the day?’, with the options ‘yes/no/more info'. Lastly, for the fourth aspect the user has to answer the question 'Do you consent to the company behind the app using and storing your data?', again with the options 'yes/no/more info'.

Furthermore, the design should be such that it is easy for the user to change consent settings at any time in order to enable the user to rescind consent once given,48An anonymous reviewer has rightly pointed out that the possibility for users to rescind their consent once given could interfere with the goal of keeping users persistent in order for users to achieve changes in their behaviour or attitude. This is indeed a valid point, but I want to emphasize that it is of primary importance to ensure that users voluntarily consent to their use of a PT (and if they do so no longer, they should be able to rescind their consent once given). Only when consent is reasonably assured, the goal of keeping users persistent should be obtained. which is necessary in order to ensure O'Neill's requirement that a person is never coerced.

However, unintended outcomes of a PT can complicate consent. Often, it is difficult to predict in advance whether a PT will have the intended effect on a user’s behaviour, because the outcome might differ depending on the individual characteristics of each user. Think, for example, of the unintended outcome that the GlowCap had on Miriam's sense of powerlessness, creating a pathogenic source of vulnerability for her. The taxonomy of vulnerability calls attention to the fact that various sources of vulnerability affect the overall result of an intervention. In addition, the effects of especially dispositional vulnerabilities and situational sources of vulnerability, although potentially influential, may not be immediately visible. To forestall this problem of unintended outcomes as well as possible, it is important to provide a user with easy ways of rescinding consent once given.

Furthermore, a person can change over time. In the context of PTs, this is especially plausible because that is exactly what the technology is after: a change in attitude and behaviour. This could lead to a situation in which a person no longer consents to one of the four aspects of the PT, in contrast to her position when she started to use the technology. Again, in order to forestall this problem, it is crucial to provide the user with easy ways of rescinding consent once given.

Furthermore, it is important to keep in mind that a consent procedure may itself compound a source(s) of vulnerability, for example if the information provided in the consent process is formulated in a way that is hard to understand for people with little education. They might find the information that is provided to them hard to understand but at the same time feel the need to make a decision, thereby consenting (or not consenting) to something that they do not quite understand. The need to respond to something that is not quite understood could cause the uncomfortable feeling of falling short. In such a case, the consent procedure compounds a pathogenic vulnerability. In order to forestall this, the information provided in a consent procedure should not only be accurate and relevant, as O'Neill has pointed out, but also easy to understand. In general, it is important that designers pay attention to the possibility that a consent procedure itself may compound vulnerabilities for (vulnerable) people, and designers should reflect upon how to forestall this.

Moreover, one could question at a more fundamental level whether we need consent requirements with regard to PTs at all. Could someone not just stop using a PT the minute she senses she is no longer using it voluntarily? After all, unlike the decision to undergo surgery, the decision to use a PT seems easily reversible.

This assumption is based on a misunderstanding, especially with regard to vulnerable people, who might become more easily dependent as a result of either psychological factors or external pressure on the workings of a PT. Being dependent on a PT could have the result that it is difficult to 'just stop' using the PT when you are no longer using it voluntarily.

Consider the example of a vulnerable person who suffered a severe burnout and is using a PT that aims to reintegrate her into the workplace. For this person, it might be difficult to stop using the technology because that might complicate her reintegration trajectory, although she no longer voluntarily ascribes to the PT. Or consider the fact that employers often provide for the use of PTs to employees. If an employee decided to stop using the technology, it might be the case that she herself has to repay the costs to her employer, leaving her with a hard penalty for opting out.

Lastly, a serious concern with any form of consent procedure (to a greater or lesser extent, depending on what the procedure looks like) is that users do not really read the terms and conditions that a technology or service provides.49I want to thank an anonymous reviewer for pointing out this concern. Instead, Bernal has pointed out that users generally scroll down a long page of writing and click 'OK' at the end to confirm that they agree to the terms and conditions, without actually reading them. Bernal calls this ‘click‐wrap consent’, which is close to meaningless.50Bernal, P. (2014). *Internet privacy rights: Rights to protect autonomy (Cambridge Intellectual Property and Information Law)*. Cambridge, U.K.: Cambridge University Press. p. 36. A similar point is made by Custers, who points out that people generally do not read privacy policies. And *if* people do read them, they often do not understand them, or they lack the required knowledge to make an informed decision, or they are not offered the choice that reflects their preferences.51Custers, B. (2016) Click here to consent forever: Expiry dates for informed consent. *Big Data & Society, 3*(1). This is a serious complication with consent procedures that is not easy to solve. However, the consent procedure inspired by O'Neill that has been advocated in this article is a relatively easy and user‐friendly one such that users can readily read and understand the terms, and can provide and possibly rescind consent when they want to.

To conclude this section, we need design requirements for PTs that ensure genuine consent by accounting for all four aspects of PTs, namely the intended behavioural outcomes, the persuasive tools, the type of individual interactions between the PT and user, and the use and storage of user data by the company behind the PT. This requires giving users a limited amount of accurate and relevant information and providing them with user‐friendly ways to extend this amount, thereby checking that they are not manipulated. It also requires providing easy ways of rescinding consent once given, thereby ensuring that users are not coerced.

## 5 CONCLUDING REMARKS

I began this article by arguing that, while PT poses ethical challenges in general, it is especially important to pay attention to the challenges it poses for vulnerable people. Vulnerability is a complicated concept that is both an ontological condition of our humanity and highly context‐specific. To better understand vulnerability and how to take it into account in the design of PTs, I used the taxonomy of Mackenzie, Rogers and Dodds. This taxonomy enables us to identify (a) the wrong or harm a person is vulnerable to, (b) the source of this vulnerability, and (c) the suitable safeguards that are needed in response. I subsequently highlighted two ethical concerns that arise when designing PTs for vulnerable people: taking into account users' needs and interests, and securing user autonomy. I argued that taking into account users' interests is crucial in technology design, but it poses challenges with regard to vulnerable users. Future research is needed on interest‐elicitation tools specifically for people with vulnerabilities. With regard to autonomy, I argued that it is crucial to have a notion of consent that is suitable in the domain of PT in order to ensure that instances of manipulation and coercion are excluded from PTs. Such a notion of consent needs to ensure respect for user autonomy with regard to all four aspects of PT, as well as providing easy ways of rescinding consent once given. Only if these two ethical concerns are carefully taken into account in the design and implementation of PTs for health and wellbeing will these technologies be suitable for vulnerable people.

## CONFLICT OF INTEREST

The author declares no conflict of interest.

